# A Cyberbullying Media-Based Prevention Intervention for Adolescents on Instagram: Pilot Randomized Controlled Trial

**DOI:** 10.2196/26029

**Published:** 2021-09-15

**Authors:** Emily R Kutok, Shira Dunsiger, John V Patena, Nicole R Nugent, Alison Riese, Rochelle K Rosen, Megan L Ranney

**Affiliations:** 1 Brown-Lifespan Center for Digital Health Providence, RI United States; 2 Department of Behavioral and Social Sciences Brown University Providence, RI United States; 3 Department of Psychiatry and Human Behavior Brown University Providence, RI United States; 4 Department Pediatrics and Medial Science Alpert Medical School of Brown University Providence, RI United States; 5 Hasbro Children’s Hospital Providence, RI United States; 6 Center for Behavioral and Preventive Medicine at The Miriam Hospital Providence, RI United States; 7 Department of Emergency Medicine Alpert Medical School of Brown University Providence, RI United States

**Keywords:** cyberbullying, adolescents, mobile application, messaging, brief interventions, social media, recruitment, mobile phone

## Abstract

**Background:**

Between 15% and 70% of adolescents report experiencing cybervictimization. Cybervictimization is associated with multiple negative consequences, including depressed mood. Few validated, easily disseminated interventions exist to prevent cybervictimization and its consequences. With over 97% of adolescents using social media (such as YouTube, Facebook, Instagram, or Snapchat), recruiting and delivering a prevention intervention through social media and apps may improve accessibility of prevention tools for at-risk youth.

**Objective:**

This study aims to evaluate the feasibility and acceptability of and obtain preliminary outcome data on IMPACT (Intervention Media to Prevent Adolescent Cyber-Conflict Through Technology), a brief, remote app-based intervention to prevent and reduce the effect of cyberbullying.

**Methods:**

From January 30, 2020, to May 3, 2020, a national sample of 80 adolescents with a history of past-year cybervictimization was recruited through Instagram for a randomized control trial of IMPACT, a brief, remote research assistant–led intervention and a fully automated app-based program, versus enhanced web-based resources (control). Feasibility and acceptability were measured by consent, daily use, and validated surveys. Although not powered for efficacy, outcomes (victimization, bystander self-efficacy, and well-being) were measured using validated measures at 8 and 16 weeks and evaluated using a series of longitudinal mixed models.

**Results:**

Regarding feasibility, 24.5% (121/494) of eligible participants provided contact information; of these, 69.4% (84/121) completed full enrollment procedures. Of the participants enrolled, 45% (36/80) were randomized into the IMPACT intervention and 55% (44/80) into the enhanced web-based resources groups. All participants randomized to the intervention condition completed the remote intervention session, and 89% (77/80) of the daily prompts were answered. The retention rate was 99% (79/80) at 8 weeks and 96% (77/80) at 16 weeks for all participants. Regarding acceptability, 100% (36/36) of the intervention participants were at least moderately satisfied with IMPACT overall, and 92% (33/36) of the participants were at least moderately satisfied with the app. At both 8 and 16 weeks, well-being was significantly higher (*β*=1.17, SE 0.87, *P*=.02 at 8 weeks and *β*=3.24, SE 0.95, *P*<.001 at 16 weeks) and psychological stress was lower (*β*=−.66, SE 0.08, *P*=.04 at 8 weeks and *β*=−.89, SE 0.09, *P*<.001 at 16 weeks) among IMPACT users than among control group users. Participants in the intervention group attempted significantly more bystander interventions than those in the control group at 8 weeks (*β*=.82, SE 0.42; *P*=.02).

**Conclusions:**

This remote app-based intervention for victims of cyberbullying was feasible and acceptable, increased overall well-being and bystander interventions, and decreased psychological stress. Our findings are especially noteworthy given that the trial took place during the COVID-19 pandemic. The use of Instagram to recruit adolescents can be a successful strategy for identifying and intervening with those at the highest risk of cybervictimization.

**Trial Registration:**

ClinicalTrials.gov NCT04259216; http://clinicaltrials.gov/ct2/show/NCT04259216.

## Introduction

### Background

American adolescents’ access to smartphones has increased from 73% in 2015 to 95% in 2018 [1]. Correspondingly, adolescents report increases in web activity, with 45% reporting that they are on the web *almost constantly* in 2018 compared with 24% in 2015 [1]. This increased exposure to electronic devices and social media increases the chances of an adolescent experiencing cybervictimization, defined as aggression or bullying by means of computers, cellphones, other electronic devices, and the internet [2]. Adolescent cybervictimization is related to depressive symptoms, suicidality, posttraumatic stress symptoms, alcohol and other drug use, physical peer violence, and dating violence [3-10]. Anywhere from 15% to 70% of adolescents have reported cybervictimization (through texting, Instagram, Facebook, or other social media), with the percentage increasing in recent years [11-20]. In 2018, approximately 60% of adolescents aged 13-17 years across the United States reported having experienced at least one of the six types of abusive web behaviors in the past year, which included offensive name-calling; spreading of false rumors; receiving unsolicited explicit images or having explicit images of them shared without their consent; incessant inquiries regarding where they are, what they are doing, and who they are with by someone other than a parent; and physical threats [21]. Racial and ethnic minority youth and youth identifying as LGBTQ+ are at a higher risk of cybervictimization [22,23]. Research aimed at both reducing cyberbullying experiences and fostering resilience in response to cyberbullying can have a significant impact on the adolescent mental health.

There are several school-based interventions to reduce cybervictimization. Some use long and intensive in-person sessions plus electronic content [24,25], some are educational simulation video games [26], and several include web-based informational sites [27]. Schools are, however, addressing numerous competing goals, with challenges in including time-intensive cyberbullying interventions in the already packed curricula. School-based interventions also assume student attendance, with some of the highest-risk students evidencing inconsistent attendance [24,28,29].

Technology-based prevention interventions have several benefits, including cost-effectiveness and scalability. The limitations of these interventions generally revolve around participants’ engagement with the technology provided [27]. In our team’s prior work, we have developed, iteratively refined, and piloted technology-augmented prevention interventions for victims of cyberbullying to be delivered during a clinic visit [28]; although these interventions had high acceptability, identifying participants in person was challenging. These findings have been recently corroborated [30].

Social media has the potential to both identify and deliver interventions to adolescents at the highest risk of cybervictimization and its consequences. Among this age group, social media use, especially Instagram, is high [21]. It is likely easier to identify at-risk groups using web-based recruitment strategies [31-34] than using in-person strategies. Social media has reduced barriers to participation (eg, transportation and stigma) [31] and offers the potential for improved honesty and increased comfort for participants [27,35]. Moreover, web-based recruitment and intervention studies can adhere to physical distancing regulations related to the COVID-19 pandemic. Interventions conducted during pandemics or forced isolations are vitally important given that many youth report worsening mental health and increased anxiety, which can have lasting effects [36].

### Objectives

This study, IMPACT (Intervention Media to Prevent Adolescent Cyber-Conflict Through Technology), was a pilot randomized controlled trial of a remote-only, 2-part intervention (brief remote video intervention plus 8-week app-based automated messaging)—built off our prior clinic-based work—to reduce the consequences of cybervictimization and improve bystander intervention behaviors [28]. Our primary hypothesis was that it would be feasible and acceptable to recruit and deliver this technology-augmented intervention purely remotely (through social media and app). The exploratory goal of this study is to examine whether this intervention would increase bystander interventions and decrease cybervictimization and postvictimization consequences, specifically social support, psychological stress, and well-being.

## Methods

### Recruitment

From January 30,2020, to May 3, 2020, targeted Instagram advertisements were used to recruit a national sample of adolescents for a screening survey. These findings have recently been corroborated [30]. Briefly, the Facebook Business Manager platform was used to present Instagram advertisements to our target audience of adolescents, who were aged 13-17 years, English speaking, and residing in the United States. Through these advertisements, adolescents were invited to complete a brief screening survey, administered using REDCap (Research Electronic Data Capture) [37], which confirmed the basic demographics (age and US residence) and eligibility requirements for the randomized controlled trial (own a cellphone and cybervictimization in the past 12 months) [38]. Eligible participants were then asked to complete a web-based assent form and six assent-related comprehension questions (eg, “If you agree to be in this study, what are we asking you to do?”). Participants that completed the assent and demonstrated comprehension of the study (by answering all six questions correctly within three attempts) were asked to complete a short baseline survey (see measures in Table 1). Contact information was verified for eligible enrolled youth through a phone call.

**Table 1:** Outline of measures and timepoints.

Measure			Screening		Baseline		8 Weeks		16 Weeks
**Cybervictimization**									
	UNH^a^ Internet Safety Education Survey: cybervictimization incidents	✓		✓		✓		✓	
	UNH Internet Safety Education Survey: bystander solutions			✓		✓		✓	
	UNH Internet Safety Education Survey: bystander efficacy			✓		✓		✓	
	YBS^b^	✓				✓		✓	
	CVEI^c^ scale	✓				✓		✓	
**Accessibility, feasibility, and usability**									
	UEX^d^					✓			
	SUS^e^					✓			
**Demographics**									
	National Study for Adolescent Health	✓							
	GenIUSS^f^ group	✓							
	Network for LGBT^g^ Health Equity at the Fenway Institute	✓							
**Mental well-being**									
	WHO-5^h^ Well-Being Index			✓		✓		✓	
	PROMIS-PS^i^			✓		✓		✓	
	PROMIS-PA^j^			✓		✓		✓	
	MSPSS^k^			✓		✓		✓	
**Other violence**									
	CADRI^l^			✓		✓		✓	
	YRBS^m^			✓		✓		✓	
	IBS^n^			✓		✓		✓	

^a^UNH: University of New Hampshire.

^b^YBS: Ybarra Bullying Scale.

^c^CVEI: Cybervictimization Emotional Impact.

^d^UEX: User Experience Questionnaire.

^e^SUS: System Usability Scale.

^f^GenIUSS: Gender Identity in US Surveillance.

^g^LGBT: lesbian, gay, bisexual, transgender.

^h^WHO-5: World Health Organization-Five.

^i^PROMIS-PS: Patient-Reported Outcomes Measurement Information System-Psychological Stress.

^j^PROMIS-PA: Patient-Reported Outcomes Measurement Information System-Positive Affect.

^k^MSPSS: Multidimensional Scale of Perceived Social Support.

^l^CADRI: Conflict in Adolescent Dating Relationships Inventory.

^m^YRBS: Youth Risk Behavior Survey.

^n^IBS: Illinois Bully Scale.

**Figure 1:**
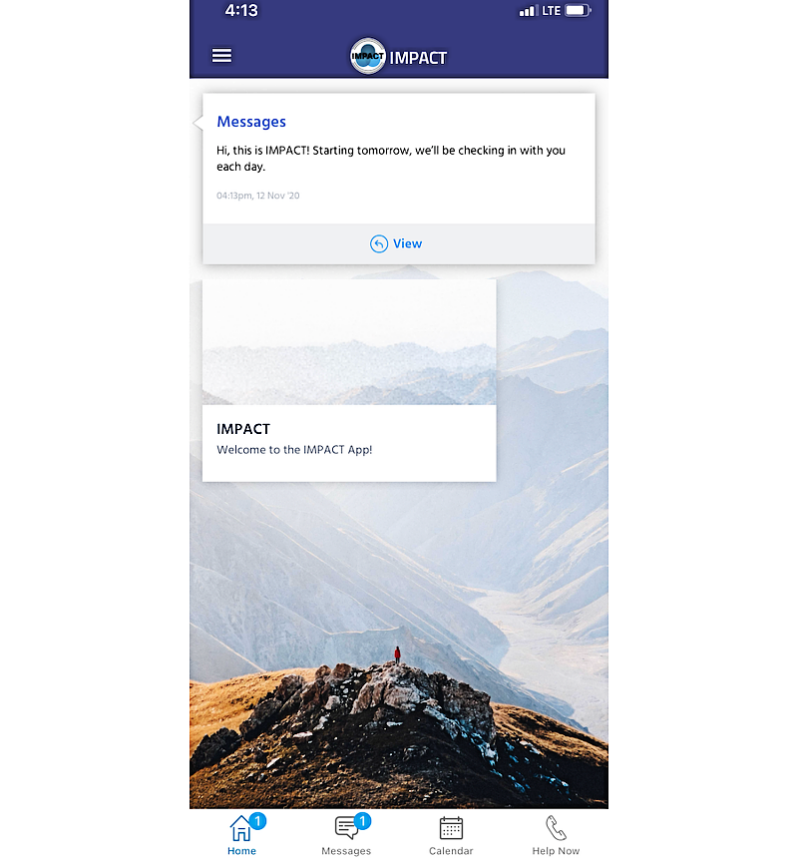
Screenshot of the IMPACT (Intervention Media to Prevent Adolescent Cyber-Conflict Through Technology) “app”.

Teens were then randomized using REDCap into either the previously piloted cyberbullying prevention intervention app (*app*; Figure 1) or into an enhanced web-based resource (control) group [28]. The randomization scheme was generated based on a permuted block randomization procedure with small, random-sized blocks. Group assignment was stratified by age and gender to ensure equal allocation of participants in each condition across the stratum. Participants and the recruiting research assistant (RA) were made aware of their group assignments; however, coinvestigators and outcome assessors were blinded. A waiver of parental consent was requested in accordance with the Common Rule [39] and in accordance with the recommendations from the Society of Adolescent Medicine [40]. This study received approval from the Rhode Island Hospital Institutional Review Board and is registered at Clinical Trials (NCT04259216).

### Study Design

#### Intervention

The purpose of the intervention was to increase self-efficacy for bystander interventions against cyberbullying and to reduce the psychosocial consequences of cybervictimization. These interventions were iteratively developed and refined through adolescent feedback [28]. Adolescents in the intervention group received a brief (15-20 minutes) remote PowerPoint intervention, delivered by a bachelor’s level RA with training in motivational interviewing (MI) principles and basic cognitive behavioral therapy (CBT) techniques [28,41-43]. The RA guided the participants through the PowerPoint, asking open-ended questions and engaging the participant in brief discussions revolving around the thoughts-feelings-actions triangle and bystander responses [42,43]. The brief intervention session covered 3 primary strategies in cybervictimization prevention and intervention: (1) Learn how to handle it, once it has happened to you or someone else; (2) Keep it from happening (sometimes impossible to do); and (3) Help stop it when you see it. Participants were asked to give an example of a prior experience with cybervictimization and then walked through how their thoughts, feelings, and actions are all connected and how changing one can influence the others. The interventionist then instructed the participants about the goal-setting procedures. They were then onboarded to the app (developed by JourneyLabs) and assisted with downloading the app onto their phone. The fully automated app delivered a daily query, at the time of the participants’ choice, asking the following: (1) “How are you feeling in general today (1=really bad and 5=great)?” and (2) “Any drama or conflict online today? (Reply ‘yes’ or ‘no’).” On the basis of the participants’ responses as well as their baseline characteristics, the participants then received an automated, tailored intervention message. There were 8 weeks of structured intervention messages, based on MI, CBT, and bystander intervention methodologies, delivered through these daily messages. Participants also had the option of requesting additional messages at any time if they were feeling *Happy*, *Sad*, *Angry* or *Stressed*, through a part of the app labeled *on-demand mood messages*. These on-demand messages were available 24 hours a day, and respondents could request as many messages as they needed.

Participants were informed that their responses were not monitored in real time and if they wished for immediate assistance, they should call a family member; a friend; 911; or the National Suicide Prevention Lifeline, the number to which was embedded into the *Help Now* tab on the app (Figure 1). We also had an institutional review board–approved crisis protocol for participants with multiple days of negative messages. The crisis protocol was never activated throughout the course of this pilot study.

Before the launch of the study, the RA was extensively trained on MI and CBT approaches (including personalized goal setting and normative feedback, cognitive restructuring techniques, and behavioral activation) as well the study rationale and crisis management procedures; they also completed role-playing exercises, as per our prior studies, to confirm expertise in intervention delivery [44].

#### Control

Adolescents in the control group participated in an RA-conducted study orientation phone call and received an enhanced web-based resource packet, which was discussed with the participants by the RA, providing a variety of websites, phone numbers, and other universal resources for cyberbullying, dating violence, sexual health, and mental health.

### Follow-Up Procedures

All participants completed a baseline, 8-week, and 16-week self-report survey. At 8 weeks, participants in the intervention group were additionally asked to complete a 30-minute semistructured remote interview using Google Meet.

### Measures

Refer to Table 1 for an outline of the primary and secondary measures and the timepoints at which they were assessed.

#### Cybervictimization

Cyberbullying questions used to determine eligibility were adapted from the University of New Hampshire Internet Safety Education Survey cybervictimization questions, including victimization incidents, bystander solutions, and bystander efficacy subscales [45]. The cybervictimization incidents 5-item subscale measures actual behaviors in response to cyberbullying (ie, did adolescents respond to their own victimization in ways that are productive). Responses were given on a 5-point scale that ranged from *0=Never* to *4=7 or more times*. The bystander solutions 11-item subscale measures bystander responses (ie, did adolescents intervene in *others*’ victimization in productive ways). Response options were mostly Yes, or No. One item was given on a 5-point scale that ranged from *0=Never* to *4=7 or more times,* and if the participant endorsed 1 or more times to the prior question, they were asked to choose from a list of bystander responses. Finally, the 13-item bystander efficacy subscale measures intended behaviors if they were to witness cybervictimization: self-efficacy in bystander response, acceptance of responsibility for response, knowledge about how to intervene, and intent to use a bystander response. Responses were given on a 5-point Likert scale that ranged from *1=Strongly Disagree* to *5=Strongly Agree*. Cybervictimization impairment during normal life events was measured using the Ybarra Bullying Scale [46]. The emotional impact of cybervictimization on participants was measured using the Cybervictimization Emotional Impact 7-item scale [47]. Responses were given on a 5-point Likert scale that ranged from *0=Not at all* to *4=Extremely*. All were measured at past 12-month (baseline only) and past-2-month (if endorsing one or more incidents at baseline and at both follow-ups) timeframes. All scales were summed, with higher sums corresponding to higher numbers of experiences or behaviors.

#### Acceptability, Feasibility, and Usability

Acceptability, measured using the User Experience Questionnaire, a 15-item self-report measure created for the study, was defined as 80% agree or strongly agree ratings. Feasibility was defined as 80% completion of the intervention protocol, including daily responses to app-based surveys. Usability was measured using the System Usability Scale (SUS), a 10-item scale used to assess the ease and appropriateness of the use of mobile intervention components [48]. Responses were given on a 5-point Likert scale that ranged from *1=Strongly Disagree* to *5=Strongly Agree*. As is the standard for this scale, responses were summed and evaluated as a continuous measure.

#### Demographics

To measure age, race, ethnicity, and socioeconomic status, selected questions from the National Study for Adolescent Health were asked during the screening survey [38]. Gender was measured using a question from the Gender Identity in the US Surveillance Group [49]. Sexual orientation was measured using a question from the Network for Lesbian, Gay, Bisexual, Transgender Health Equity at the Fenway Institute [50].

#### Mental Well-being

The World Health Organization-Five Well-Being Index was used to measure overall well-being in the past 2 weeks [51-53]. The World Health Organization-Five assesses current mental well-being using five statements on a 5-point Likert scale ranging from *0=At no time* to *5=All of the time*. The Patient-Reported Outcomes Measurement Information System (PROMIS)-Psychological Stress is a 4-item subscale that assesses cognitive-perceptual disruption, feeling overwhelmed, and perceived lack of control to manage one’s own life [54,55]. The PROMIS-Positive Affect is a subscale that assesses in-the-moment positive and rewarding affective experiences over the past 7 days [56]. Response options for both PROMIS scales were given on a 5-point Likert scale ranging from *0=At no time* to *5=All of the time.* The Multidimensional Scale of Perceived Social Support was used to assess social support systems from friends, family, and significant others [57-59]. Responses were given on a 7-point Likert scale that ranged from *1=Very Strongly Disagree* to *4=Half and half* to *7=Very Strongly Agree.* As per standard practice, all scales were scored as sums and used as continuous measures.

#### Other Violence

The Conflict in Adolescent Dating Relationships Inventory was used to assess the history of physical dating violence in adolescents who have been in a relationship during the given time points. This scale uses sum scoring, with higher scores indicating greater amounts of abuse [60]. Experiences with physical assault were measured using a single item from the Youth Risk Behavior Survey, which is consistent with prior work [61]. In-person bullying was measured using 7 items from the victim and bully subscales of the Illinois Bully Scale [62,63]. Response options for all 3 of these scales that measure *other violence* were on a 5-point Likert scale ranging from *0=Never* to *4=7 or more times.* All these scales were assessed at past 12-month (baseline only) and past 2-months (if endorsing one or more incidents answer at baseline and at both follow-ups) timeframes.

### Semistructured Interviews

To further assess acceptability, feasibility, and usability, a trained RA conducted semistructured interviews with all participants in the intervention arm. Participants were asked open-ended questions regarding the content of the app message intervention, remote intervention, and any logistical changes or improvements (Multimedia Appendix 1). Interviews lasted between 12 and 36 minutes (average length: 23 minutes, SD 5.67) and were digitally recorded. Overarching categories for a framework matrix–guided analysis were created by the interviewer (a bachelor’s-level RA) and a senior coinvestigator. Within each category, answers were further divided by sentiment codes (positive, negative, neutral, or suggesting change). Summaries were abstracted directly from the recordings in a framework matrix format by a team of 3 RAs. The first 10% (4/36) of interviews were double coded for content or sentiment by an RA and the interviewer; thereafter, 10% (3/32) of codes were double checked for accuracy. All abstractions were verified by the interviewer while listening to the recordings. Key quotes were transcribed into the aforementioned categories by the RA [64,65]. Any discrepancies were discussed and resolved by the RA, interviewer, and coinvestigator. The abstracted results were reviewed by the study team, and themes from the summaries were developed by the senior coinvestigator and the study team.

### Statistical Analysis

Descriptive statistics for the entire sample were calculated; between-group differences were examined using two-tailed *t* tests for continuous variables and chi-square tests for categorical variables and nonparametrics as appropriate.

The primary outcomes of feasibility and acceptability were examined in both conditions using descriptive statistics, including measures of self-reported satisfaction with the intervention, as well as objective retention and adherence rates. Between-group differences in the SUS scores were examined using two-tailed *t* tests.

The exploratory outcomes of interest were changes from baseline in cybervictimization (measured by the University of New Hampshire Internet Safety Education Survey [45]); bystander interventions; and postvictimization consequences, specifically social support, psychological stress, and well-being. A secondary set of exploratory outcomes included other types of violence. Using a series of longitudinal mixed-effects models, we assessed the effects of condition on these outcomes. The outcome at each follow-up was simultaneously regressed on condition, time, condition×time, baseline value, and sex (a covariate chosen a priori). The models included subject-specific intercepts to adjust for repeated measures over time within the participants. All analyses were conducted on the intent-to-treat sample (all randomized participants included in the analysis). Mixed-effects models take a likelihood approach to estimation, thus making use of all available data without directly imputing missing outcomes. The significance level was set at .05 a priori, and all analyses were conducted using SAS 9.3. As this was a pilot study, no power analysis was performed before the initiation of the study.

## Results

### Overview

**Figure 2:**
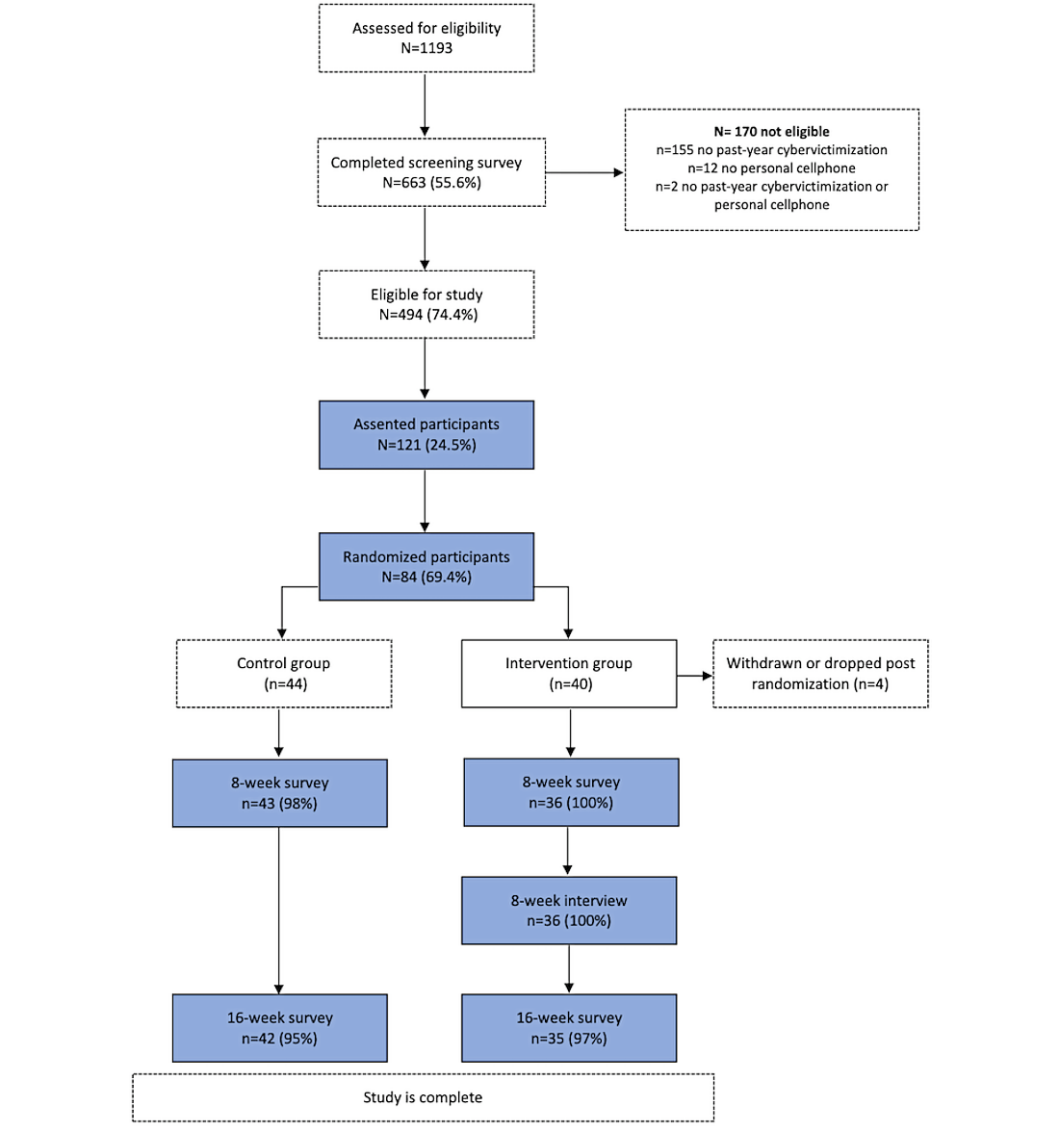
Participant recruitment flow through the IMPACT (Intervention Media to Prevent Adolescent Cyber-Conflict Through Technology) pilot trial.

Recruitment continued for 907.5 nonconsecutive hours of Instagram advertisements until the target goal of 80 enrolled participants (Figure 2) was achieved [30]. During this period, 1193 screening surveys were conducted. Of the 663 participants who completed the screening survey, 494 (74.4%) met the eligibility criteria, 121 (24.5%) completed the assent form (37 participants either did not respond to the contact information verification calls or did not pass the verification process), and 84 (69.4%) were randomized into the intervention (n=36) or control groups (n=44). Four participants randomized to the intervention group were either withdrawn or dropped before receiving any intervention materials. A full description of the study flow is presented in Figure 2.

On average, participants were 15.3 (SD 1.35) years old and predominantly identified their gender as female (47/80, 59%) and non-Hispanic (70/80, 88%). More than two-thirds of the participants reported that Instagram was their most commonly used social media platform (Table 2). There were no between-group differences in baseline demographics and psychosocial or baseline bullying scores (*P*>.05 for all).

The CONSORT (Consolidated Standards of Reporting Trials)-eHealth checklist (V 1.6.1) of this study can be found in Multimedia Appendix 2 [1,3-23,27,28,30,36,66-68].

**Table 2:** Participants’ baseline demographics, cybervictimization, history of physical violence, and overall mental health (N=80).

Characteristic		Intervention (n=36)	Control (n=44)
Age (years), mean (SD)		15.28 (1.32)	15.36 (1.38)
**Self-reported gender, n (%)**			
	Cisgender female	22 (61)	25 (57)
	Cisgender male	10 (28)	11 (25)
	Transgender, nonbinary, or “other”	4 (11)	8 (18)
**Sexual orientation, n (%)**			
	Not straight	16 (47)	28 (67)
**Race, n (%)**			
	White	24 (67)	30 (68)
	Black	1 (3)	5 (11)
	Multiracial	5 (14)	7 (16)
	Other	1 (4)	2 (5)
**Ethnicity, n (%)**			
	Non-Hispanic	31 (86)	39 (89)
**SES^a^, n (%)**			
	Lower income	12 (33)	15 (34)
Prior use of mental health services^b^, n (%)		16 (44)	22 (50)
**Region, n (%)**			
	Northeast	4 (11)	7 (16)
	Southeast	10 (28)	7 (16)
	Midwest	6 (17)	12 (27)
	West	11 (30)	10 (23)
	Southwest	5 (14)	8 (18)
**Most common social media, n (%)**			
	Instagram	23 (64)	31 (71)
**Prior violence exposure, mean (SD)**			
	Past-year cybervictimization^c^	6.00 (4.76)	4.86 (4.70)
	Past-year physical fights^d^	0.11 (0.32)	0.07 (0.33)
	Past-year dating violence^e^	1.47 (1.31)	1.05 (0.22)
	Past-year in-person bullying^f^	7.86 (4.86)	6.70 (4.71)
**Psychological well-being, mean (SD)**			
	Well-being^g^	12.66 (4.03)	11.80 (4.65)
	Positive affect^h^	12.67 (3.39)	12.02 (2.93)
	Psychological stress^i^	14.19 (3.48)	14.09 (4.05)
	Perceived social support^j^	45.23 (9.34)	40.02 (11.97)

^a^SES: socioeconomic status.

^b^Seeing a counselor or being hospitalized in a psychiatric facility in the past 12 months.

^c^University of New Hampshire Internet Safety Education Survey number of cybervictimization incidents subscale.

^d^Youth Risk Behavior Survey fighting subscale.

^e^Conflict in Adolescent Dating Relationships.

^f^Illinois Bully Scale.

^g^World Health Organization-Five.

^h^Patient-Reported Outcomes Measurement Information System-Positive Affect.

^i^Patient-Reported Outcomes Measurement Information System-Psychological Stress.

^j^Multidimensional Scale of Perceived Social Support.

### Feasibility and Acceptability

All participants who scheduled remote interventions completed the session and downloaded the app. We had over 98% retention rate at 8 and 16 weeks for intervention and over 95% for control groups, representing the proportion of participants who completed the follow-up survey at that time point. All participants randomized into the intervention group completed an 8-week remote interview. There were 3587 responses to the daily mood surveys (out of 4032 potential responses), resulting in a daily response rate of 89%. A total of 71 on-demand messages from 19 unique participants were requested (happy=36, sad=15, stressed=12, and angry=8). Taken together, the intervention was considered feasible based on a priori benchmarks.

All participants reported on the User Experience Questionnaire that they were at least moderately satisfied with IMPACT overall, with 92% (33/36) of intervention participants reporting that they were at least moderately satisfied with the IMPACT app. When asked about the frequency of messaging, 86% (31/36) of participants endorsed that it was *just right*; the remaining 14% (5/36) felt that the messaging was too frequent. When asked if they would recommend IMPACT, 86% (68/80) responded positively. There were no significant between-group differences in the SUS scores.

### Exploratory Outcomes

A full description of the adjusted treatment effects on the cybervictimization and bystander scores at 8 and 16 weeks are presented in Table 3. Although point estimates of the overall prevalence of personal cybervictimization were in the expected direction, there was no significant between-group effect at 8 or 16 weeks. However, a significant treatment effect was observed on the number of solutions tried for combating personal cybervictimization, with intervention participants reporting a significantly higher number of strategies at 8 weeks (*β*=.82, SE 0.42; *P*=.02). Although not significant, the point estimate was similar at 16 weeks. Finally, intervention participants had significantly higher bystander self-efficacy and intention to help others (*β*=2.65, SE 1.32; *P*=.04) at 8 weeks than participants in the control group.

Table 4 presents adjusted treatment effects of exploratory outcomes of well-being and other secondary violence outcomes. Results indicate significant treatment effects on well-being at 8 and 16 weeks such that intervention participants had significantly better overall well-being (*β*=1.17, SE 0.87, *P*=.02 at 8 weeks and *β*=3.24, SE 0.95, *P*<.001 at 16 weeks), decreased stress (*β*=−.66, SE 0.08, *P*=.04 at 8 weeks and *β*=−.89, SE 0.09, *P*<.001 at 16 weeks), and higher social support (*β*=3.50, SE 2.02, *P*=.049 at 16 week, with no differences at 8 weeks) than control participants. Intervention participants reported significantly fewer physical fights at 8 weeks than control participants (*β*=−.60, SE 0.28, *P*=.01) but no change in dating violence or in-person bullying.

**Table 3:** Treatment effects on exploratory outcomes of cyberbullying and bystander interventions in the past 2 months (comparing baseline to 8 weeks to 16 weeks).

Exploratory outcome	8 Weeks		16 Weeks	
	*β*^a^ (SE)	*P* value^b^	*β* (SE)	*P* value
Number of experiences of cybervictimization^c^	−3.25 (3.32)	.35	−.83(2.26)	.72
Interference of cybervictimization with normal life^d^	−.31 (0.45)	.49	−.14 (0.54)	.79
Emotional effect of cybervictimization^e^	.18 (1.26)	.89	.51 (1.48)	.73
Solutions tried for combating cybervictimization^f^	*.82 (0.42)^g^*	*.02*	.74 (0.56)	.10
Frequency of observed cyberbullying^f^	.21 (0.24)	.38	.10 (0.17)	.57
Frequency of bystander intervention^f^	.49 (0.45)	.28	.43 (0.49)	.39
Efficacy and intention for bystander intervention^h^	*2.65 (1.32)*	*.04*	1.82 (1.44)	.21

^a^Unstandardized regression coefficient.

^b^*P* values are obtained from the model of intervention versus control on scores at 8 weeks and 16 weeks controlling for baseline and sex.

^c^University of New Hampshire Internet Safety Education Survey number of cybervictimization incidents subscale.

^d^Ybarra Bullying Scale summed, 3-item.

^e^Cybervictimization Emotional Impact.

^f^University of New Hampshire Internet Safety Education Survey bystander solutions.

^g^Italics indicates *P*<.05.

^h^University of New Hampshire Internet Safety Education Survey bystander self-efficacy and intention to help others.

**Table 4:** Secondary outcomes of general mental health, well-being, and other violence (comparing baseline to 8 weeks and to 16 weeks).

Secondary outcomes	8 Weeks			16 Weeks	
	*β*^a^ (SE)	*P* value^b^	*β* (SE)		*P* value
Well-being^c^	*1.17* (*0.87*)^d^	*.02*	*3.24 (0.95)*		*<.001*
Psychological stress^e^	−*.66 (.08)*	*.04*	−*.89 (0.09)*		*<.001*
Positive affect^f^	.61 (0.60)	.32	.55 (0.69)		.42
Perceived social support^g^	−.45 (1.59)	.78	*3.50 (2.02)*		*.05*
Dating violence in the past 2 months^h^	.38 (0.28)	.18	1.15 (1.07)		.29
In-person bullying in the past 2 months^i^	3.07 (3.00)	.41	1.66 (2.70)		.60
Number of physical fights^j^	−*.60 (0.28)*	*.01*	−.07 (0.04)		.10

^a^Unstandardized regression coefficient.

^b^*P* values are obtained from model of intervention versus control on scores at 8 weeks and 16 weeks controlling for baseline and sex.

^c^World Health Organization-Five.

^d^Italicized values indicate statistical significance (*P*<.05).

^e^Patient-Reported Outcomes Measurement Information System-Psychological Stress.

^f^Patient-Reported Outcomes Measurement Information System-Positive Affect.

^g^Multidimensional Scale of Perceived Social Support.

^h^Conflict in Adolescent Dating Relationships.

^i^Illinois Bully Scale.

^j^Youth Risk Behavior Survey fighting subscale, 1-item.

### User Experience

Semistructured interviews (Multimedia Appendix 1) were conducted with the intervention group only (n=36). The interview material was coded into 5 general themes: message content and tone, remote intervention content, usability or helpfulness of the app, preference between app-based messaging and text-based messaging, and recommending the program (Table 5). All intervention participants had positive comments on the message content and tone, and some participants had useful suggestions about future changes. Almost all participants had positive comments regarding the remote intervention content; negative reactions mostly focused on the length of the presentation. Approximately one-fourth of the participants suggested improvements to the app interface. Three-quarters of the participants said that they preferred an app-based intervention (compared with other technology-based intervention modalities such as text messaging or the web). They said that the app has the potential to hold more resources, it is separate from personal conversations with friends, and it sends them reminders to answer the survey if they forget. Finally, all participants said that they would recommend the program to friends. Two participants provided a caveat that they would not recommend the program to adults in general or to adolescents who do not check their notifications often.

**Table 5:** Representative quotes from the qualitative interviews (n=36).

Theme	Quote	Participant
Message content and tone	“I actually really liked the messages and I felt like especially when I was like at a friend’s house or just not really focusing or like really remembering, the messages helped me look back at what the whole point of this was and helped me remember all the helpful tricks...I really enjoyed the content because especially at my school, they have touched on it very lightly. But it was not something I fully understood like thoroughly and I feel like all the content really helped me get a better understanding.”	14-year-old female
Remote intervention content	“...Mostly the little class or whatever you would call it at the beginning was most helpful for me, but the links were still good...I felt like I got the most tools from like that lesson we did at the beginning and the messages were more a reminder that I skimmed through to remember what we talked about.”	17-year-old male
Usability or helpfulness of the app	“It was a nice appearance...a nice aesthetic...Instead of the tabs [at the bottom of the app labeled ‘Home, Messages, Calendar, and Help now’] you could have push buttons on the Home-screen kind of like the apps on your phone, and then maybe at the top where it says welcome to the app you could put the messages and notification board there.”	17-year-old male
App-based messaging	“I like the app better [than other formats] because it was a separate thing and I feel like with text messages you would respond different...I wouldn’t have responded as much to text messages”	16-year-old male
Recommending the program	“Yes, I have already tried to recommend it before I love it a lot...[I recommended it to] several of my friends especially ones that I find getting down more easily or seem to get in a lot of arguments online I recommended it to them.”	15-year-old nonbinary

## Discussion

### Principal Findings

In this study, we demonstrated that IMPACT, an entirely remote intervention, is feasible, is acceptable, and may be effective in improving bystander intervention and well-being among adolescents with a history of cybervictimization. Our analysis is encouraging given the increasing social isolation of youth during COVID-19, reported increases in web-based and in-person violence during the pandemic, and difficulty in disseminating school-based programs. It is possible that a positive change can be made using the same device that is used for cyberbullying. These results add to a growing body of work showing that it is possible for technology-augmented interventions to reduce violence and improve mental health among at-risk adolescents, and provides a strong rationale for the public health imperative of disseminating these remote and technology-based interventions [26,27,44].

The IMPACT intervention and study design were not just feasible but also highly engaging. The daily response rate to the 2-question survey was 89%, and study retention was close to 100% up to 4 months after enrollment. Many web-based interventions struggle with engagement and retention [27,66]. The reasons for our higher-than-average engagement likely include our design process, in which we iteratively refined our app based on participant feedback [28], and our use of 2-way communication, which has been shown to increase retention and engagement [67]. It may also reflect youths’ willingness to participate in research to help others.

Despite not being powered for effect sizes, we observed a significant, positive effect at 8 weeks on intervention group participants’ efficacy in and intention to use cyberbullying bystander interventions and in the number of strategies tried in response to their own cybervictimization, compared with the control group. This finding is remarkable, given that other studies have reported nonsignificant effects on increasing behavioral changes related to cybervictimization [25]. These significant effect sizes may reflect participants’ repeated exposure to bystander intervention content and modeling of bystander interventions during the 8-week intervention. The lack of effect at 16 weeks may reflect a small sample size or the need for continuous exposure, and it should be further investigated with boosters or enhanced interventions. The lack of change in the prevalence of cybervictimization was expected given the period of measurement (16 weeks), lack of intervention with those who perpetrated bullying, and possible increased awareness of experiences of cybervictimization due to study participation.

Participants in the intervention group also showed significant increases in overall mental well-being and decreases in psychological stress compared with the control group. Other studies have shown that during pandemics or forced isolations, many youth report worsening mental health and likely increased anxiety, which can extend for months to years [36]. The increase in well-being and decrease in psychological stress in the intervention group is, therefore, particularly noteworthy given that all follow-up time points occurred during the government-enforced quarantine of the COVID-19 pandemic. These findings correspond with our prior work with text-message-based interventions to reduce physical violence and improve mental health among at-risk adolescents [44], showing that technology-augmented interventions can help increase adolescents’ overall well-being.

A high percentage of participants reported being LGBTQ+ and having a low socioeconomic status. These groups are at the highest risk of cybervictimization and poor mental health [22,23]. During the COVID-19 pandemic, a time in which in-person support was decreased for all, web-based recruitment, and delivery of interventions to these youth are critically important. Our findings suggest that web-only recruitment and interventions can be used strategically to reach the youth at the highest risk of cybervictimization. Future work should also examine the relative diversity of recruitment and efficacy of interventions for remote-only secondary prevention interventions such as IMPACT versus in-person universal prevention interventions in schools.

Overall, the participants provided positive feedback regarding the remote intervention and app-based messaging program. Notably, all participants in the intervention group said that they would recommend the program to friends or other adolescents experiencing cyberbullying. During the interview process, it became clear that participants were highly familiar with the structure and format of popular social media apps; therefore, they were able to provide informative comments on improvements to the intervention app. This acceptability of an app-based intervention provides further evidence that a mobile app and brief remote session can be an effective format for adolescent behavioral interventions [68].

Future refinement and dissemination are indicated.

### Limitations

Despite the high percentage of participants that reported a low socioeconomic status and identified as a gender and sexual minority, the overall percentage of eligible participants enrolled in the study was low; our participants may not, therefore, be fully representative of the national adolescent population. Semistructured interviews were conducted by the same RA who guided the participants through the brief intervention. As rapport was built through these interactions, participants may have felt more comfortable sharing their beliefs about the acceptability and feasibility of the study; however, rapport may also have led to social desirability bias in responses. As we used a single social media tool (Instagram) for recruitment, we may have missed youth who primarily used other forms of social media. Although Instagram is one of the most popular social media sites among adolescents [21], other social media sites such as Snapchat and TikTok might lead to a more inclusive representation. Importantly, we began recruitment on January 30, 2020, before COVID-19 was declared a national pandemic. Due to these environmental changes, the baseline characteristics of participants enrolled before the national emergency status may be slightly different than those enrolled after March 2020.

### Conclusions

Overall, this study suggests that remote recruitment and enrollment in an app-based intervention is highly acceptable and feasible and may be effective in improving well-being, increasing bystander intervention, and growing coping strategies among adolescents who experience cybervictimization. Although the prevalence of cybervictimization was not changed by the intervention, this was not expected because of the focus on increasing bystander effects. Finally, this study suggested that adolescents who are at high risk of cybervictimization can be successfully reached through web-based recruitment methods.

## Appendix

Multimedia Appendix 1 Semistructured interview questions.

Multimedia Appendix 2 CONSORT-eHEALTH checklist (V 1.6.1).

## Abbreviations

CBT: cognitive behavioral therapy
CONSORT: Consolidated Standards of Reporting Trials
IMPACT: Intervention Media to Prevent Adolescent Cyber-Conflict Through Technology
MI: motivational interviewing
PROMIS: Patient-Reported Outcomes Measurement Information System
RA: research assistant
REDCap: Research Electronic Data Capture
SUS: System Usability Scale
