# Supplementary material for: A Cyberbullying Media-Based Prevention Intervention for Adolescents on Instagram: Pilot Randomized Controlled Trial
Source: JMIR Ment Health. 2021 Sep 15;8(9):e26029. doi: 10.2196/26029 (PMC8482167; doi:10.2196/26029)
Supplement: Multimedia Appendix 1 [file mental_v8i9e26029_app1.docx]

**Multimedia Appendix 1. Semistructured interview questions.**

Semi-structured interview questions asked of intervention participants only at 8-weeks:

**A. FEEDBACK on the intervention content**

*Intent: The goal of section is to get global feedback on the messages and remote intervention.*

1. To start, can you tell me your experiences of getting the IMPACT messages?
   1. Did you like getting the messages?
      1. What did you like/dislike about them?
         1. Probe: Content (topics, tone)
         2. Probe: Format (frequency, length, and duration)
   2. Did you ever use any of the on-demand mood messages [ANGRY, SAD, STRESSED, HAPPY]?
      1. Are there any other crave words we should consider using in the program?
   3. Mood Tracker?
   4. PowerPoint/video?
   5. Click on links?
   6. Did you ever share the messages with a friend?
   7. Did you have any concerns about privacy?
2. **Remote session:** Before you started getting the messages, way back when you first agreed to participate in this program, we talked over the phone and reviewed some materials
   1. Is there anything from that session that you remember particularly well?
   2. Is there anything from that session that you particularly liked or disliked?
   3. What did you think about the process of enrolling through Instagram?
      1. What did you think about the ads/what would you change?

**B. FEEDBACK on the mobile app**

*Intent: In this section, we will collect data about the participant's concerns and expectations related to JourneyLabs^TM^, their willingness to use the app, and what they would want to see in the app.*

1. How often did you use the app?
   1. If not, why not?
   2. If so, what did you think about it?
2. Did you find the app helpful?
   1. What parts (if any) were not helpful?
   2. What parts (if any) did you find helpful?
3. What was the greatest challenge in using it?
4. What did you think of the features, how it looks?
   1. Were any parts of it confusing or hard to use?
   2. Did the notifications work well for you?
5. If you were given the choice between regular text messaging and receiving the messages through the app, which would you have preferred?
6. LiveText?
7. Thinking back, is there anything you would have changed about the app?
   1. Are there any additional services that you wish had been included?
8. Would you recommend this experience to other people you know?
   1. Who would you recommend it to?
9. Is there anyone you would not recommend the experience to?

**C. THOUGHTS about direct measurement of cyberbullying**

*The intent of this section is to obtain participants’ views on the direct measurement of cyberbullying. We will be asking participants to allow us to download their social media (Instagram) interactions prior to the start of the interview. This has been done in other studies, but not in the context of cyberbullying. We are particularly interested in hearing what influenced participants’ decision to let us download their data or not, and how we can increase the chance that participants will agree to it in future aims.*

- - - 1. We asked if you would be willing to allow us to download an anonymous version of your social media (Instagram) interactions, in order to figure out whether cyberbullying has been happening.

Probe: Why did you or did you not agree to this?

Probe: What influenced your decision?

Probe: [*if participant did not agree*] What would INCREASE the chance of your being willing to do this in the future?

Probe: What were your concerns about this when we asked you?

Is there anything you did not want us to download or that you wanted us to delete after seeing it downloaded?

**D. CLOSING SECTION**

1. Is there ANYTHING ELSE we should know about your experience with the IMPACT program? Do you have any tips on how to help us to help other teens, both to prevent cyberbullying and to deal with it better?
